# What are the principles that underpin integrated care?

**DOI:** 10.5334/ijic.1884

**Published:** 2014-11-27

**Authors:** Lourdes Ferrer, Nick Goodwin

**Affiliations:** International Journal of Integrated Care; International Journal of Integrated Care

In previous editorials, it has been argued that the study of integrated care has focused perhaps overmuch on understanding the complex and multi-dimensional nature of integrated care as a process and less on its role as a ‘movement for change’ in terms of fundamentally challenging the current and future design of care systems [1]. For example, whilst it is commonly perceived that integrated care should be fundamentally person-centred (i.e. that practical approaches to integrated care should be characterised by services that are responsive to people's holistic needs), it is rare to listen to those who think integrated care should also involve people and communities as co-producers of care [2].

Another growing debate is whether disease-specific programmes, for example those co-ordinating care for patients with a chronic illness such as diabetes, should really be considered as ‘integrated care’. Over the years, integrated care has become synonymous with approaches to address the fragmentation and lack of coordination and continuity of care across time, place and discipline traditionally encountered by populations with various chronic and disabling conditions [3]. Yet, there is evidence to suggest that a disease-based focus has not necessarily embraced the necessary holistic, or people-centred, philosophy that integrated care represents. This is manifest, for example, in the separate organisation and delivery of vertical disease programmes (such as for TB, HIV and malaria) and the failure of many chronic care programmes in tackling both physical and mental health co-morbidities or related factors such as frailty.

In reflecting on these debates, it becomes clear that integrated care is not just challenged by the difficulties in defining what we mean by the term, but also that there has never been developed a set of principles that sets out the underlying philosophy or set of key beliefs that should underpin the term. Indeed, the lack of a core set of values is probably one of the reasons there is such variation in the way people interpret its meaning.

Over the past year, the International Foundation for Integrated Care has been working with the World Health Organisation in supporting the development of their Global Strategy on People-Centred and Integrated Health Services [4]. A part of IFIC's work has been to develop a set of core guiding principles in order for future reforms of health systems to be grounded to a common set of goals and aspirations. By reflecting on the views and comments from international stakeholders and partners involved in the development of the Strategy, 16 core principles were identified (see Box 1).

It should be recognised that these suggested core principles have been derived in the context of a global strategy seeking to provide countries with ways in which they might overcome existing fragmentations in care delivery in order to be in a better position to achieve the aspirations of universal health coverage. The principles are intentionally broad to enable them to reflect the realities of deploying integrated care strategies in different country contexts and settings. However, it would be quite feasible for these principles to be extended by, for example: stressing the importance of payment methods that provide economic incentives which support integrated delivery; recognising the importance of political and managerial leadership in taking action to transform care; or the importance of shared care records with patients.

Developing a list of principles begins to set out a set of common criteria through which to frame the aspirations of the integrated care movement and so present a common philosophy through which to judge current and future efforts. The two questions this editorial asks are: does integrated care need a set of principles and are the principles we suggest above the right ones? We invite readers to write to the Editor with your views and/or join the debate by visiting the IFIC LinkedIn discussion at: https://www.linkedin.com/groupItem?view=&gid=8193942&type=member&item=5933356201977876482&trk=groups_most_popular-0-b-ttl&goback=%2Egmp_8193942.

## Note

The WHO Strategy on People-Centred and Integrated Health Services will be presented in Edinburgh at the 15th International Conference on Integrated Care (ICIC15) on 25–27 March 2015. More details available at: http://www.integratedcarefoundation.org/conference/15_annual.

## Figures and Tables

**Box 1.**
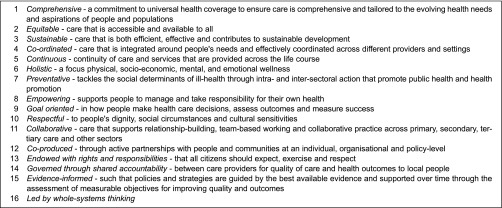
Suggested core principles guiding people-centred and integrated health services
